# Faunistic notes on Cryptophagidae and Latridiidae of Talassemtane National Park, Western Rif, Morocco, with the description of a new species (Coleoptera, Cucujoidea)

**DOI:** 10.3897/zookeys.668.11347

**Published:** 2017-04-12

**Authors:** José Carlos Otero, Yousra Benyahia, Hervé Brustel

**Affiliations:** 1 Departamento de Zoología y Antropología Física, Facultad de Biología, 15782 Santiago de Compostela, Spain; 2 Laboratoire d’Ecologie et Environnement, Faculté des Sciences Ben M’sik, Université Hassan II-Casablanca, avenue Cdt Driss El Harti, BP 7955, Sidi Othman-Casablanca, 20000 Casablanca, Morocco; 3 Université de Toulouse, École d’Ingénieurs de Purpan, INPT, UMR Dynafor 1201, 75 voie du TOEC, 31076 Toulouse cedex 3, France

**Keywords:** Coleoptera, Cryptophagidae, Dienerella (Cartoderema) talassemtana sp. n., Latridiidae, Morocco, Rif, Talassemtane National Park

## Abstract

In order to contribute to the knowledge of beetles (Coleoptera) of the mountainous region of Morocco, Talassemtane National Park (Western Rif, Chefchaouen district, Morocco) was surveyed. This is an exceptional protected area of the Mediterranean Intercontinental Biosphere Reserve (RIBM). The checklist was made using different traps combined with active periodical searches during 2013–2015. A total of 153 beetles belonging to 19 species from four subfamilies (Cryptophagidae: Cryptophaginae and Atomariinae; Latridiidae: Latridiinae and Corticariinae) was collected. Dienerella (Cartoderema) talassemata, a new species (Coleoptera: Latridiidae) was compared to other morphologically related species. One species is recorded for the first time for North Africa; three species are new records for Morocco. In addition, amongst the species listed, three are endemic to Morocco: *Dienerella talassemtana* **sp. n**., *Caenoscelis humifera* and *Dienerella besucheti*.

## Introduction

Created in October, 2004, Talassemtane National Park (PNTLS) is located in the western area of the calcareous ridge of the Rif mountain range. The park constitutes a unique territory containing natural landscapes of great heritage value at a national level. Its position at the boundary line between Europe and Africa, its climatic and geological characteristics, and its paleogeographic history, have given rise to unique fauna and flora. As such, the national park is included in the Mediterranean Intercontinental Biosphere Reserve (RBIM), intended for the conservation of the most emblematic natural areas of northern Morocco and southern Spain. Talassemtane National Park harbours more than 750 plant species (56 endemic) belonging to 103 families (Meda 2008). It also lodges about 40 species of mammals and more than 100 species of birds; reptiles and amphibians are represented by about 30 species with a rate of endemism reaching 27% (Meda 2008). Aquatic macro-invertebrates comprise 180 species of which 48 are endemic. Obviously, the invertebrates of PNTLS have been less studied than plants and vertebrates, with only a few of studies to date on Diptera, Simuliidae (Belqat et al. 2001), ants (Taheri et al. 2014), and water beetles (Benamar et al. 2011). To tackle the lack of information on invertebrates of Moroccan protected areas and to enrich the list of the Rifian entomofauna and especially that of PNTLS, a study of the Coleoptera was carried out. This study was developed within the framework of the Agronomic Research Program for Development (PRAD). In addition to the checklist, the objective of this study is to detect species related to the forest stands in order to establish a management policy that aims to improve their conservation. In this paper, we treat only the families Cryptophagidae and Latridiidae.

Cryptophagidae is a moderately large group of small-sized beetles (1–6 mm long) containing more than 1,000 described species belonging to approximately 50 genera. Cryptophagidae has a worldwide distribution, and as indicated by Crowson (1980), some groups have amphipolar distribution. Both adults and larvae are commonly found on mold, fungi, under bark as well as in decaying vegetation and nests of social hymenoptera, birds and mammals (Otero et al. 2001; Lyubarsky and Perkovsky 2011). Latridiidae is a moderately large family with approximately 500 species which is represented in all major biogeographic regions. They are minute (1-3 mm) and often live in moldy stored food and decomposed plant materials. They seem to be spore feeders (Pal and Ghosh 2007).

## Materials and methods

### Study site

**Figure 1.**
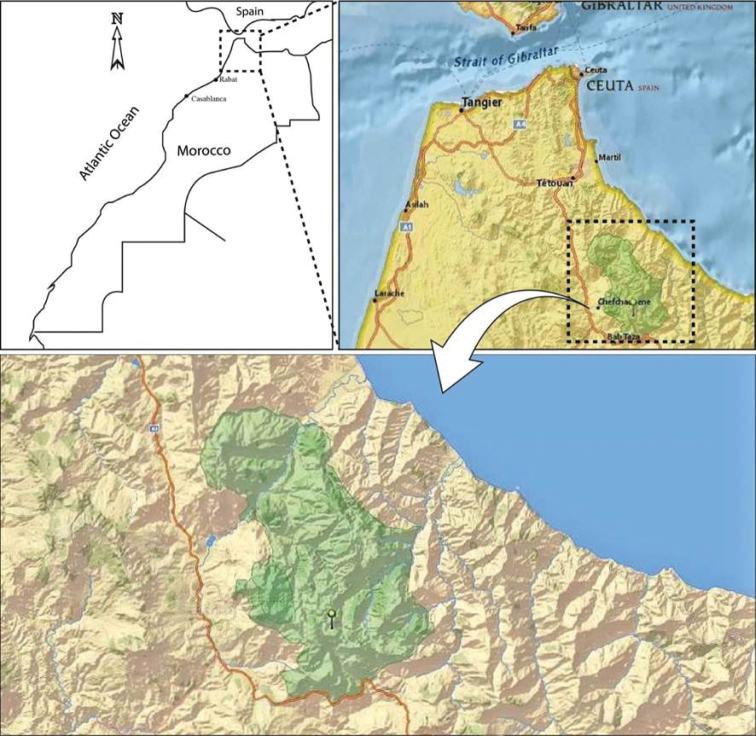
Map of Morocco showing the location of Talassemtane National Park. The green cropped area indicates the limits of the Park.

PNTLS covers an area of 64,601 ha. Two-thirds of the park are located within the province of Chefchaouen and one-third belongs to the province of Tetouan (Fig. 1). It is mainly formed by the southern area of the Rifian calcareous ridge and includes the highest summits of western Rif. The climate of PNTLS is characterized by being the wettest zone of North Africa. The park is characterized by two climax forest stands, formed by an endemic and relict fir species, *Abies maroccana* Trab. The Talassemtane fir forest is the largest and extends over 2,300 ha. Our survey was carried out in pure fir (*Abies alba*) forests (in the highest zones), as well as in fir-oak (*Quercus ilex*) forests.

### Methods

Terminology and measurements of new species follow an earlier paper (Otero 1997). Morphological structures were measured under a Leica M205C stereomicroscope equipped with an analysis system Application Suite.

### Data collection

The records of trap contents were performed once every 15 days, for 7 months (April–October), during 2013 and 2014. Window flight traps, and in particular the multidirectional PolytrapTM, were used as they are probably the best suited for inventorying saproxylophagous beetles in temperate forest. The output of these traps and their selective power are rather enlightening, as far as the capture of Coleoptera is concerned, compared to other arthropods (Bouget and Brustel 2009). The multidirectional window flight traps were primed with ethanol, which serves as an attractant for numerous saproxylophagous beetles (Bouget and Brustel 2009). Two other types of traps were used in the checklist; pitfall traps were installed at ground level to intercept ground fauna, and yellow-coloured traps were used in open environments to attract flower-dwelling species. All traps were installed in 20 unsystematic plots in the fir grove.

Active searching was done during April 2013, August 2013, October 2013, June 2014, September 2014 and November 2015, most often during the installation of the protocol or the placement of traps. Many active methods were then used such as barking dead trees, raising stones, beating or sifting.

### Abbreviations

**L** length;

**WL** width/length ratio;

**E** eccentricity of the eyes, which is calculated as width/half of the length (width is measured across the widest part of a line joining the anterior and posterior limit of the eye; length is the maximum length of the eye);

**L** length in dorsal view;

**W** width;

**Ø** diameter.

**CBT** coll. H. Brustel, Toulouse, France.

**COCC** coll. O. Courtin, Castres, France.

**CWR** coll. W. Rucker, Neuwied, Germany.

**MNHN** Museum National d’Histoire Naturelle, Paris, France.

**USCO** Universidad de Santiago de Compostela, coll. J.C. Otero, Santiago de Compostela, Spain.

## Results

In total, 153 beetles belonging to 19 species from four subfamilies (Cryptophagidae: Cryptophaginae and Atomariinae; Latridiidae: Latridiinae and Corticariinae) were collected. Dienerella (Cartoderema) talassemata represents a new species (Coleoptera: Latridiidae) for the Palaearctic Region. One species is recorded for the first time for the north of Africa: *Cryptophagus cylindrellus* C. Johnson, 2007. Three species are new records for Morocco: *Cryptophagus pallidus* Sturm, 1845; *Cryptophagus uncinatus* Stephens, 1830 and Atomaria (Atomaria) nigripennis (Kugelann, 1794). In addition, amongst the species listed, two others are endemic to Morocco: *Caenoscelis humifera* Esser, 2008 and Dienerella (Cartoderema) besucheti Vincent, 1994.

### Family Cryptophagidae

#### Subfamily Cryptophaginae

##### Caenoscelis humifera

Taxon classificationAnimaliaColeopteraCryptophagidae

Esser, 2008

Caenoscelis humifera Esser, 2008: 7

###### Examined material.

Morocco, Rif, Talembote, Sapinière de Talassemtane, 10-13.XI.2015, 1 ex (leg. H. Brustel). Mts Rif, Al-Hoceima, Torres-de-Alcatá, Steilküste, 27.XII.2001, 1 ex; Jbel Tazzeka, Taza, Gorges du Zireg, 5.I.2002, 1 ex (leg. Esser)

###### Distribution.

Endemic to Morocco (Esser 2008; Otero 2013).

##### Cryptophagus cylindrellus

Taxon classificationAnimaliaColeopteraCryptophagidae

C. Johnson, 2007

Cryptophagus cylindrellus C. Johnson, 2007: 66

###### Examined material.

Morocco, Rif, Sapinière de Talassemtane, 22. V.2014, 1 ex.; 15.VI.2014, 1 ex; 19.VI.2014, 2 exx; 3.X.2014, 3 exx (leg. H. Brustel).

###### Distribution.

Throughout Europe and Turkey (Johnson et al. 2007; Otero 2013).

First mention for North Africa.

##### Cryptophagus dentatus

Taxon classificationAnimaliaColeopteraCryptophagidae

(Herbst, 1793)

Cryptophagus dentatus (Herbst, 1793:15)

###### Examined material.

Morocco, Rif, Sapinière de Talassemtane, 19.VII.2012, 1 ex; 4. V.2013, 1 ex; 9. V.2013, 1 sp; 24. V.2013, 1 ex; Talembote, Sapinière de Talassemtane, 30. X.2015, 1 ex; 10-13.XI.2015, 3 exx (leg. H. Brustel).

###### Distribution.

Europe, North Africa, Central Asia and North America (Johnson et al. 2007; Otero and Johnson 2013).

##### Cryptophagus jakowlewi

Taxon classificationAnimaliaColeopteraCryptophagidae

Reitter, 1888

Cryptophagus jakowlewi Reitter, 1888: 424

###### Examined material.

Morocco, Rif, Sapinière de Talassemtane, 4.V.2013, 8 sp.; 9.V.2013, 4 exx; 26.IX.2013, 1 sp; 31.X.2013, 4 exx; 30.IV.2014, 2 exx; 15.VI.2014, 7 exx; 19.VI.2014, 24 exx; 18.X.2014, 1 sp; 15.X.2016, 2 exx; Talembote, Sapinière de Talassemtane, 7.V.2015, 1 ex; 10-13.XI.2015, 8 exx (leg. H. Brustel).

###### Distribution.

Europe, North Africa, Caucasus, Asia Minor, Central Asia and Eastern Siberia (Johnson et al. 2007; Otero 2013).

##### Cryptophagus pallidus

Taxon classificationAnimaliaColeopteraCryptophagidae

Sturm, 1845

Cryptophagus pallidus Sturm, 1845: 69

###### Examined material.

Morocco, Rif, Talembote, Sapinière de Talassemtane, 10-13.XI.2015, 3 exx (leg H. Brustel).

###### Distribution.

Throughout Europe, North Africa (Algeria, Lebanon and Tunisia), Iran, Israel, Lebanon and Turkey (Johnson et al. 2007; Otero 2013).

First mention for Morocco.

##### Cryptophagus pubescens

Taxon classificationAnimaliaColeopteraCryptophagidae

Sturm, 1845

Cryptophagus pubescens Sturm, 1845: 103

###### Examined material.

Morocco, Rif, Sapinière de Talassemtane, 15.VI.2014, 1 ex (leg H. Brustel).

###### Distribution.

Europe, Caucasus and North Africa (Johnson et al. 2007; Otero 2013).

##### Cryptophagus punctipennis

Taxon classificationAnimaliaColeopteraCryptophagidae

C.N.F. Brisout de Barneville, 1863

Cryptophagus punctipennis Brisout de Barneville, 1863: 63

###### Examined material.

Morocco, Rif, Talembote, Sapinière de Talassemtane, 10-13.XI.2015, 5 exx (leg. H. Brustel).

###### Distribution.

Cosmopolitan species (Johnson et al. 2007; Otero 2013).

##### Cryptophagus scanicus

Taxon classificationAnimaliaColeopteraCryptophagidae

(Linnaeus, 1758)

Cryptophagus scanicus (Linnaeus, 1758: 357)

###### Examined material.

Morocco, Rif, Talembote, Sapinière de Talassemtane, 7.V.2015, 2 exx (leg. H. Brustel).

###### Distribution.

Holarctic (Johnson et al. 2007; Otero 2013).

##### Cryptophagus uncinatus

Taxon classificationAnimaliaColeopteraCryptophagidae

Stephens, 1830

Cryptophagus uncinatus Stephens, 1830: 75

###### Examined material.

Morocco, Rif, Sapinière de Talassemtane, 30.IV.2014, 1 ex (leg. H. Brustel).

###### Distribution.

Throughout Europe, North Africa (Algeria) and Turkey (Johnson et al. 2007; Otero 2013).

First record for Morocco.

### Family Latridiidae

#### Subfamily Atomariinae

##### Atomaria (Atomaria) nigripennis

Taxon classificationAnimaliaColeopteraLatridiidae

(Kugelann, 1794)

Atomaria (Atomaria) nigripennis (Kugelann, 1794: 578)

###### Examined material.

Morocco, Rif, Talembote, Sapinière de Talassemtane, 10-13.XI.2015, 9 exx (leg H. Brustel).

###### Distribution.

Europe and North Africa (Tunisia) (Johnson et al. 2007; Otero 2011).

First record for Morocco.

##### Atomaria (Atomaria) pallidipennis

Taxon classificationAnimaliaColeopteraLatridiidae

Holdhaus, 1903

Atomaria (Atomaria) pallidipennis Holdhaus, 1903: 364

###### Examined material.

Morocco, Rif, Sapinière de Talassemtane, 4. V.2013, 1 sp.; 30.IV.2014, 1 ex (leg. H. Brustel).

###### Distribution.

Europe and North Africa (Johnson et al. 2007; Otero 2011).

##### Atomaria (Atomaria) pusilla

Taxon classificationAnimaliaColeopteraLatridiidae

(Paykull, 1798)

Atomaria (Atomaria) pusilla (Paykull, 1798: 295)

###### Examined material.

Morocco, Rif, Talembote, Sapinière de Talassemtane, 10-13.XI.15, 1 ex (leg. H. Brustel).

###### Distribution.

Europe, Caucasus, Mongolia, Turkey, Iran, Afghanistan, Central Asia, North Africa, Madeira and the United States (Johnson et al. 2007; Otero 2013).

#### Subfamily Latridiinae

##### Cartodere (Aridius) nodifer

Taxon classificationAnimaliaColeopteraLatridiidae

(Westwood, 1839)

Cartodere (Aridius) nodifer (Westwood, 1839: 155)

###### Examined material.

Morocco, Rif, Sapinière de Talassemtane, 19.VI.2014, 1 ex (leg H. Brustel).

###### Distribution.

Cosmopolitan species (Johnson 2007).

##### Dienerella (Cartoderema) besucheti

Taxon classificationAnimaliaColeopteraLatridiidae

Vincent, 1994

Dienerella (Cartoderema) besucheti Vincent, 1994: 77

###### Examined material.

Morocco, Rif, 12 km W Bab Berret, 35°01'06"N, 05°00'40"W, 11.IV.2013, 2 exx (det. W. Rücker) (leg. H. Brustel).

###### Distribution.

Endemic to Morocco (Johnson 2007; Vincent 1994).

##### Dienerella (Cartoderema) talassemtana sp. n.

Taxon classificationAnimaliaColeopteraLatridiidae

http://zoobank.org/69EF0F36-5247-43D4-99F0-3DC957E3AEBE

Fig. 2A–E

###### Description.

**Figure 2.**
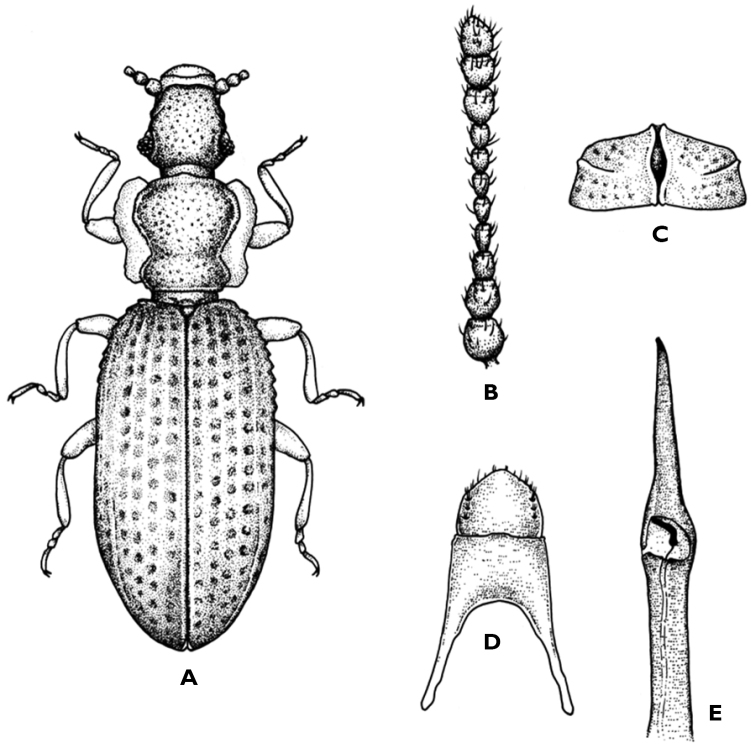
Dienerella (Cartoderema) talassemtana sp. n. **A** dorsal habitus **B** antennae **C** elytral suture **D** tergite V masculine **E** aedeagus.

Body length: 1.16–1.31 mm. Body elongated, narrow and superficially depressed. Reddish grey-brown or testaceous-brown colour; lighter appendages. Body (Fig. 2A) glabrous. Lacking metathoracic wings. **Head** rough and transverse (WL= 1.4); slightly narrower (including the eyes) than the pronotum in its anterior region. Clypeus short, as wide as the head, separated from the frons by a slightly arcuate suture and from the labrum by a concave suture. Labrum visible from top view. Temples short, oblique and visible from top view. Eyes hemispherical and slightly protruding (E=1), not surpassing the margin of the pronotum, made up of few (15 to 20) facets. Eye facets with small diameter (Ø= 6–8 µm). Antennae (Fig. 2B) short (L= 0.314 mm), reaching the pronotum constriction. Antennomere I spherical, wider and 1.4 longer than the II; subequal from III to VIII. The last three form an extended club; X as long as IX; XI truncate distally, 1.3 times longer than the previous one. **Pronotum** 1.1 times longer than wide. Anterior and posterior margins straight; lateral margins rounded. Strongly constricted in the basal third. Rough surface with marked (Ø= 10–15 µm) and thick puncturation. Tibiae with a small spine on the apex. **Elytra** together oval, elongated; 1.8 times longer than wide; rounded base. Lateral margins rounded and denticulate in their anterior half. Provided with 8 rows of large (Ø= 24–30 µm) and ordered punctures (4 dorsal and 2 lateral). Space between suture and fourth elytral interval slightly convex, not excavated behind the scutellar shield. Slight depression in the last third of the dorsal area of the elytra. Fifth striae higher, forming a curved lining that slightly separates near the middle area and joins to the suture in the posterior area; fourth striae higher in the anterior two thirds. Elytral suture bearing a rhomboid opening in the posterior area (Fig. 2E). Anterior coxae almost adjacent; intermediate separated and posterior widely separated. Tergite V masculine (Fig. 2D). **Aedeagus** (Fig. 2E). Aedeagus with very elongated apex, not widened. Internal sac as in Fig. 2E.

###### Type material.

**Morocco. Holotype** (m). Morocco, Rif, Talembote, P.N. Talassemtane, 10-13.XI.2015 (leg. H. Brustel) (USCO).

###### Paratypes.

1 m. Morocco, Rif, P.N. Talassemtane, 13.XI.2015 (leg O. Courtin), (COCC). 1 f. Morocco, Rif, Talembote, P.N. Talassemtane, 10-13.XI.2015 (leg. H. Brustel) (MNHN). 1 f. Morocco, Rif, Talembote, P.N. Talassemtane, 10-13.XI.2015 (leg. H. Brustel), (CBT). 1 f. Morocco, Rif, Talembote, P.N. Talassemtane, 10-13.XI.2015 (leg. H. Brustel), (CWR).

###### Distribution.

Morocco.

###### Etymology.

This species is named in reference to the region in which the type material was collected.

#### Differential diagnosis

In order to distinguish the species of *Dienerella* Reitter of the group *elongata* (Curtis), the following table may be useful (from Rücker 1998, modified)

**Table:** 

1	Posterior margin of tergite V in males with distinct apical widening (Fig. 3A). Lanceolate aedeagus (Fig. 3D). L=1.4–1.5mm	***D. huguettae* Vincent**
–	Posterior margin of tergite V masculine without distinct apical widening. Aedeagus with very elongated apical end	**2**
2	Posterior margin of tergite V masculine rounded (Fig. 3C, D	**3**
–	Posterior margin of the tergite V masculine slightly pointed (Figs 2D, 3B)	**4**
3	Aedeagus with very elongated and narrow apical end; armour of internal sac as in Fig. 3H. L=1.3–1.7 mm	***D. clathrata*** (**Mannerheim)**
–	Aedeagus with very elongated and narrow apical end; C-shaped armour of internal sac (Fig. 3G)	***D. besucheti* Vincent**
4	Aedeagus with elongated and spatulate apex (Fig. 3F); armour of internal sac as in Fig. 3F. L=1.2–1.4 mm	***D. separanda* (Reitter)**
–	Aedeagus with very elongated and not widened apex (Figs 2E); armour of internal sac as in Fig. 2E. L= 1.16–1.31 mm	***D. talemsattana* sp. n.**

**Figure 3.**
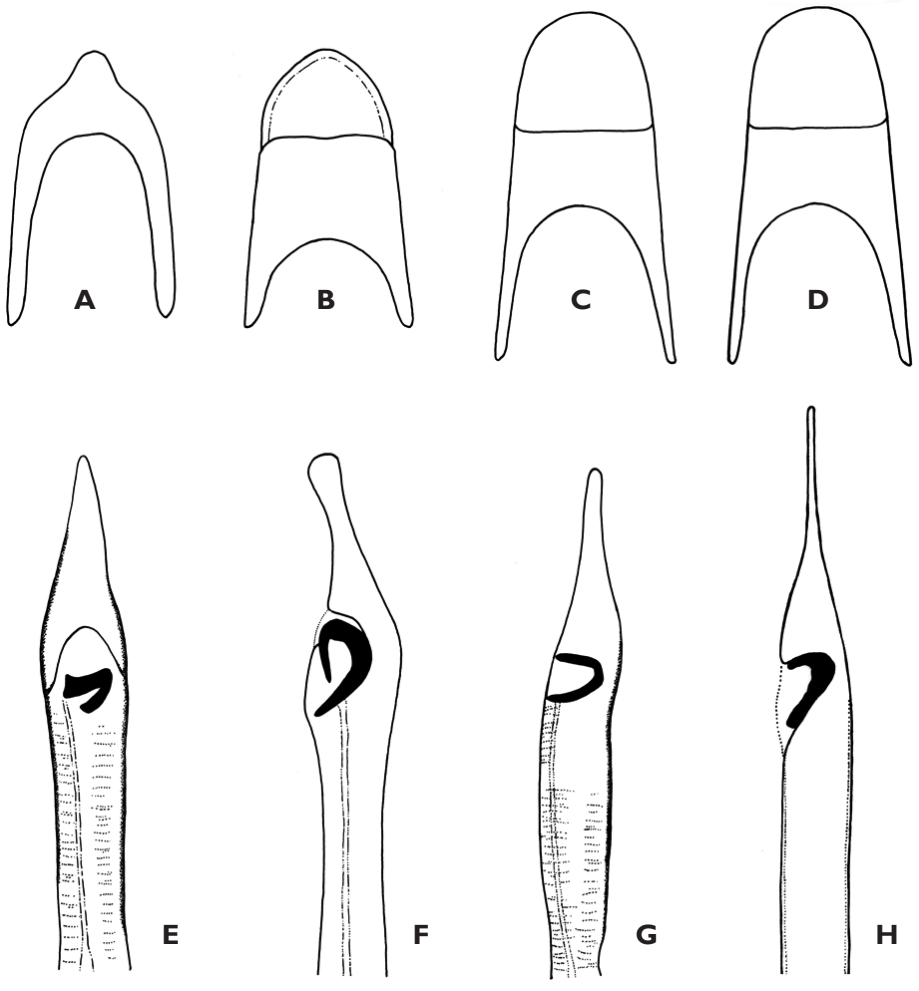
Tergite V masculine and aedeagus: **A, E** *Dienerella huguetae* Vincent **B, F** *Dienerella separanda* (Reitter) **C, G** *Dienerella besucheti* Vincent **D, H** *Dienerella clathrata* (Mannerheim).

##### Enicmus brevicornis

Taxon classificationAnimaliaColeopteraLatridiidae

(Mannerheim, 1844)

Enicmus brevicornis (Mannerheim, 1844: 102)

###### Examined material.

Morocco, Rif, Sapinière de Talassemtane, 9.IV-4. V.2013, 1 ex; 24.V-4.VI.2013, 1 ex; 4. VI-20.VI. 2013, 2 exx; 20.VI.4.VII. 2013, 1 ex; 31.VII-15. VIII.2013, 11 exx (det. W. Rücker); 15. VIII.2013, 11 exx; 31. VIII.2013, 1 ex; 10.IX-26.IX. 2013, 1 ex (det. W. Rücker); 31. X.2013, 4 exx; 10. IV-30.IV.2014, 1 ex; 30.IV.2014, 1 ex; 15.V.2014, 1 ex; 30.VI-15.VII. 2014, 1 ex (det. W. Rücker); 15.VII.2014, 1 ex; Talembote, Sapinière de Talassemtane, 10-13.XI.15, 1 ex, (leg. Y. Benyahia)

###### Distribution.

Europe, North Africa, Iran and Turkey (Johnson 2007).

##### Revelieria genei

Taxon classificationAnimaliaColeopteraLatridiidae

(Aubé, 1850)

Revelieria genei (Aubé, 1850: 333)

###### Examined material.

Morocco, Rif, 12 km W Bab Berret, 11.IV.2013, 35°01'06"N, 05°00'40"W, 11.IV.2013, 1 ex (det. W. Rücker) (leg H. Brustel).

###### Distribution.

France, Great Britain, Italy, Spain, Algeria, Morocco, Tunisia, Israel, Turkey (Johnson 2007).

#### Subfamily Corticariinae

##### Corticaria illaesa

Taxon classificationAnimaliaColeopteraLatridiidae

Mannerheim, 1844

Corticaria illaesa Mannerheim, 1844: 33

###### Examined material.

Morocco, Rif, Talembote, Sapinière de Talassemtane, 10-13.XI.2015, 1 ex (leg. H. Brustel).

###### Distribution.

Widespread but sporadic throughout the Mediterranean region (Johnson 2007).

##### Corticaria inconspicua

Taxon classificationAnimaliaColeopteraLatridiidae

Wollaston, 1860

Corticaria inconspicua Wollaston, 1860: 260

###### Examined material.

Morocco, Rif, Talembote, Sapinière de Talassemtane, 10-13.XI.2015, 1 ex, Sapinière de Talassemtane, 10.IV.2013, 1 ex (det. W. Rücker); Sapinière de Tazaot, 35°15'50"N 05°06'14"W, 1 ex (det. W. Rücker) (leg. H. Brustel).

###### Distribution.

Europe, North Africa and Cyprus (Johnson 2007).

## Conclusion

This brief survey allows us to contribute to the Moroccan faunistic checklist with some new data:

Dienerella (Cartoderema) talassemtana (Coleoptera: Latridiidae) represents a new species from Morocco and the the Palaearctic Region. In addition, amongst the species listed, two other species are apparently endemic to Morocco: *Caenoscelis humifera* Esser, 2008 and Dienerella (Cartoderema) besucheti Vincent, 1994. One species is mentioned for the first time for North Africa: *Cryptophagus cylindrellus* C. Johnson, 2007.

Three species are new records to Morocco: *Cryptophagus pallidus* Sturm, 1845; *Cryptophagus uncinatus* Stephens, 1830 and Atomaria (Atomaria) nigripennis (Kugelann, 1794).

This research effort must continue in the future to improve our knowledge on the Moroccan entomofauna. It is also especially interesting for the definition of the local biodiversity hot spot and the selection of suitable taxa to establish a red list of saproxylic beetles in Maghreb.

## Supplementary Material

XML Treatment for Caenoscelis humifera

XML Treatment for Cryptophagus cylindrellus

XML Treatment for Cryptophagus dentatus

XML Treatment for Cryptophagus jakowlewi

XML Treatment for Cryptophagus pallidus

XML Treatment for Cryptophagus pubescens

XML Treatment for Cryptophagus punctipennis

XML Treatment for Cryptophagus scanicus

XML Treatment for Cryptophagus uncinatus

XML Treatment for Atomaria (Atomaria) nigripennis

XML Treatment for Atomaria (Atomaria) pallidipennis

XML Treatment for Atomaria (Atomaria) pusilla

XML Treatment for Cartodere (Aridius) nodifer

XML Treatment for Dienerella (Cartoderema) besucheti

XML Treatment for Dienerella (Cartoderema) talassemtana

XML Treatment for Enicmus brevicornis

XML Treatment for Revelieria genei

XML Treatment for Corticaria illaesa

XML Treatment for Corticaria inconspicua

## References

[B1] Belqat B Adler P Dakki M (2001) Distribution summary of the Simuliidae of Morocco with new data for the Rif Mountains. British Simuliid Group 17: 9–14. http://www.blackfly.org.uk/downloadable/bulls16-20.pdf [accessed 13 September 2016]

[B2] Benamar L Bennas N Millan A (2011) Les coléoptères aquatiques du Parc National de Talassemtane (Nord-Ouest du Maroc). Biodiversité, degré de vulnérabilité et état de conservation. Boletín de la Sociedad Entomológica Aragonesa 49: 231–242.http://www.seaentomologia.org/Publicaciones/PDF/BOLN_49/231242BSEA49ColeopterosPNTLSMaroc.pdf [accessed 13 September 2016]

[B3] Bouget C Brustel H (2009) Inventaires entomologiques en forêt. Les pièges vitres. In: Bouget C Nageleisen LM (Eds) L’étude des insectes en forêt: méthodes et techniques, éléments essentiels pour une standardisation. Synthèse des réflexions menées par le groupe de travail «Inventaires Entomologiques en Forêt» (Inv.Ent.For.). Les Dossiers Forestiers n°19. Office National des Forets, Paris, 58–62. http://www.onf.fr/outils/medias/20100517-145700-588403/++files++/1 [accessed 13 September 2016]

[B4] Crowson RA (1980) On amphipolar distribution patterns in some cool climate groups of Coleoptera. Entomology Generalis 6: 281–292.

[B5] Esser J (2008) *Caenoscelis humifera* n. sp aus Marokko– eine neue ungeflügelte westpaläartische Art der Gattung *Caenoscelis* C.G.Thomson, 1863 (Coleoptera: Cryptophagidae). Mitteilungen des Internationalern Entomologischen Vereins 33(1–2): 7–15.

[B6] Johnson C (2007) Latridiidae. In: Löbl I Smetana A (Eds) Catalogue of Palaearctic Coleoptera, Vol. 4. Elateroidea, Derodontoidea, Bostrichoidea, Lymexyloidea, Cleroidea, Cucujoidea. Apollo Books, Stenstrup, 635–649.

[B7] Johnson C Otero JC Leschen RAB (2007) *Cryptophagidae*. In: Löbl I Smetana A (Eds) Catalogue of Palaearctic Coleoptera, Vol. 4. Elateroidea, Derodontoidea, Bostrichoidea, Lymexyloidea, Cleroidea, Cucujoidea. Apollo Books, Stenstrup, 513–531.

[B8] Lyubarsky GYu Perkovsky EE (2011) Third contribution on Rovno amber silken fungus beetles: a new Eocene species of *Cryptophagus* (Coleoptera, Clavicornia, Cryptophagidae). In: Shcherbakov DE Engel MS Sharkey MJ (Eds) Advances in the systematics of fossil and modern insects: Honouring Alexandr Rasnitsyn. ZooKeys 130: 255–261. https://doi.org/10.3897/zookeys.130.1321 10.3897/zookeys.130.1321 PMC3260764 22259281

[B9] Meda (2008) Parc National de Talassemtane: evaluation de la biodiversité et suivi des habitats. 208 pp.

[B10] Otero JC (1997) Three new species and distributional records of Micrambe C.G.Thomson, 1863 and *Cryptophagus* Herbst, 1792 (Coleoptera: Cryptophagidae) from Istrael and Turkey. Revue Suisse de Zoologie 104(1): 207–216. https://doi.org/10.5962/bhl.part.79997

[B11] Otero JC (2011) Coleoptera, Monotomidae, Cryptophagidae. In: Ramos MA et al. (Eds) Fauna Ibérica, vol. 35. Museo Nacional de Ciencias Naturales. CSIC, Madrid, 365 pp.

[B12] Otero JC (2013) Cryptophaginae (Coleoptera) de la Región Paleártica occidental. Coleopterological Monographs 4: 1–296.

[B13] Otero JC Johnson C (2013) Species of the genus *Cryptophagus* Herbst, 1792, belonging to the “*dentatus* group” (Coleoptera; Cryptophagidae) from the Western Palearctic region. Entomologica Fennica 24: 81–93.

[B14] Otero JC Johnson C Mifsud D (2001) Cryptophagids from the Maltese Islands with description of a new species of *Micrambe* Thomson (Coleoptera: Cryptophagidae). Koleopterologische Rundschau 71: 163–170.

[B15] Otero JC López MJ Rücker W (2013) Review of the *Corticaria sylvicola* group (Coleoptera: Latridiidae), with a description of two new species from the Iberian Peninsula. Annales de la Société Entomologique de France (N.S. ) 49(3): 233–239. https://doi.org/10.1080/00379271.2013.854090

[B16] Pal TK Ghosh S (2007) A new genus of Corticariinae from Nagaland, India. Annali Museo Civico Storia Naturale “G. Doria” 8(354): 1–10.

[B17] Rücker WH (1998) Una nuova specie italiana di Latridiidae: Dienerella angelinii n. sp. Annali Museo Civico di Storia Naturale “G. Doria” 7(306): 3–7.

[B18] Taheri A Reyes-Lopez JL Bennas N (2014) Contribution a l’étude de la faune myrmécologique du Parc National de Talassemtane (Nord du Maroc): biodiversité, biogéographie et espèces indicatrices. Boletín de la Sociedad Entomológica Aragonesa 54: 225–236.

[B19] Vincent V (1994) Nouvelle contribution à l’etude du genre *Dienerella* Reitter, 1911 D. besucheti espèce nouvelle du Maroc. Nouvelle Revue d’Entomologie (N.S. ) 11(1): 91–93.

